# RNA-Seq transcriptome profiling of mouse oocytes after *in vitro* maturation and/or vitrification

**DOI:** 10.1038/s41598-017-13381-5

**Published:** 2017-10-16

**Authors:** Lei Gao, Gongxue Jia, Ai Li, Haojia Ma, Zhengyuan Huang, Shien Zhu, Yunpeng Hou, Xiangwei Fu

**Affiliations:** 1 National Engineering Laboratory for Animal Breeding and Key Laboratory of Animal Genetics, Breeding and Reproduction, Ministry of Agriculture, College of Animal Science and Technology, China Agricultural University, Beijing, 100193 P.R. China; 2 Key Laboratory of Adaptation and Evolution of Plateau Biota, Northwest Institute of Plateau Biology, Chinese Academy of Sciences, Xining, 810001 P.R. China; 3 Institute of Animal Sciences, Chinese Academy of Agricultural Sciences, Bejing, 100193 P.R. China; 4 State Key Laboratory for Agrobiotechnology, College of Biological Sciences, China Agricultural University, Beijing, 100193 P.R. China

## Abstract

*In vitro* maturation (IVM) and vitrification have been widely used to prepare oocytes before fertilization; however, potential effects of these procedures, such as expression profile changes, are poorly understood. In this study, mouse oocytes were divided into four groups and subjected to combinations of *in vitro* maturation and/or vitrification treatments. RNA-seq and *in silico* pathway analysis were used to identify differentially expressed genes (DEGs) that may be involved in oocyte viability after *in vitro* maturation and/or vitrification. Our results showed that 1) 69 genes were differentially expressed after IVM, 66 of which were up-regulated. *Atp5e* and *Atp5o* were enriched in the most significant gene ontology term “mitochondrial membrane part”; thus, these genes may be promising candidate biomarkers for oocyte viability after IVM. 2) The influence of vitrification on the transcriptome of oocytes was negligible, as no DEGs were found between vitrified and fresh oocytes. 3) The MII stage is more suitable for oocyte vitrification with respect to the transcriptome. This study provides a valuable new theoretical basis to further improve the efficiency of *in vitro* maturation and/or oocyte vitrification.

## Introduction

*In vitro* maturation (IVM) of mammalian oocytes is an essential technique used in research of developmental biology and assisted reproductive technology (ART)^1^. However, oocytes matured *in vitro* are of lower quality than those matured *in vivo*. Embryos from oocytes matured *in vitro* have decreased preimplantation embryo development, low pregnancy rates and poor live birth indexes^2^. Moreover, knowledge about the low efficiency of IVM is poor compared to *in vivo* maturation, which limits the further development of IVM techniques^3^. A comparison of the different features of oocytes matured *in vitro* or *in vivo* via transcriptome analyses provides an opportunity to identify oocyte competence biomarkers. A study of human oocytes using microarrays has provided further insight into alterations that occur during maturation^4^; however, the suboptimal quality of the material (oocytes failed to fertilize) makes it difficult to interpret the conclusions. The differences in transcriptome profiles between in *vivo* and *in vitro* matured oocytes still require further investigation.

Oocyte cryopreservation can preserve excess oocytes obtained during treatment, averting repeated oocyte retrieval from the patients^5^. Oocyte cryopreservation can also preserve fertility in women who may lose their ovarian function because of surgery, cancer treatment, or premature menopause^6^ and in women who decide to postpone having children due to socioeconomic pressures^7^. In addition, human embryo cryopreservation is prohibited in some countries (e.g. Italy)^8^. Thus, for this case, oocyte cryopreservation could be the optimal choice to preserve fertility rather than embryo cryopreservation.

As an efficient and convenient method of cryopreservation, vitrification is now widely used. However, high concentrations of cryoprotective agents as well as chilling injury may affect the ultrastructure, such as the spindles and chromosomes^9^, and the mitochondrial function^10^ of the oocytes. Mitochondrial function can be reflected by the ratio of flavin adenine dinucleotide/ reduced nicotinamide adenine dinucleotide phosphate (the ratio of FAD/NAD(P)H) which is an established method for monitoring relative amounts of electron donor and acceptor in a cell. An increased ratio of FAD/NAD(P)H suggests a decreased ATP level in vitrified human metaphase II (MII, the second stage in meiosis II after prophase II) stage oocytes^10,11^, which significantly affects spindle integrity and chromosome alignment^12^. There are also other factors that may influence the cryopreservation efficiency of oocytes, in particular the development stage of the oocytes. For example, one major problem of MII stage oocytes is the sensitivity of the spindles to low temperatures and cryoprotective agents^13–15^. Theoretically, this damage can be avoided by vitrification of the germinal vesical (GV) stage oocytes, a stage when the spindle apparatus has not yet formed. However, oocytes vitrified in the GV stage must go through IVM after thawing, which may further influence oocyte competence. There are reports of babies born from oocytes vitrified at both of these two stages^16–18^; however, the molecular mechanism underlying which stage is more suitable for oocyte vitrification is still unclear.

**Figure 1:**
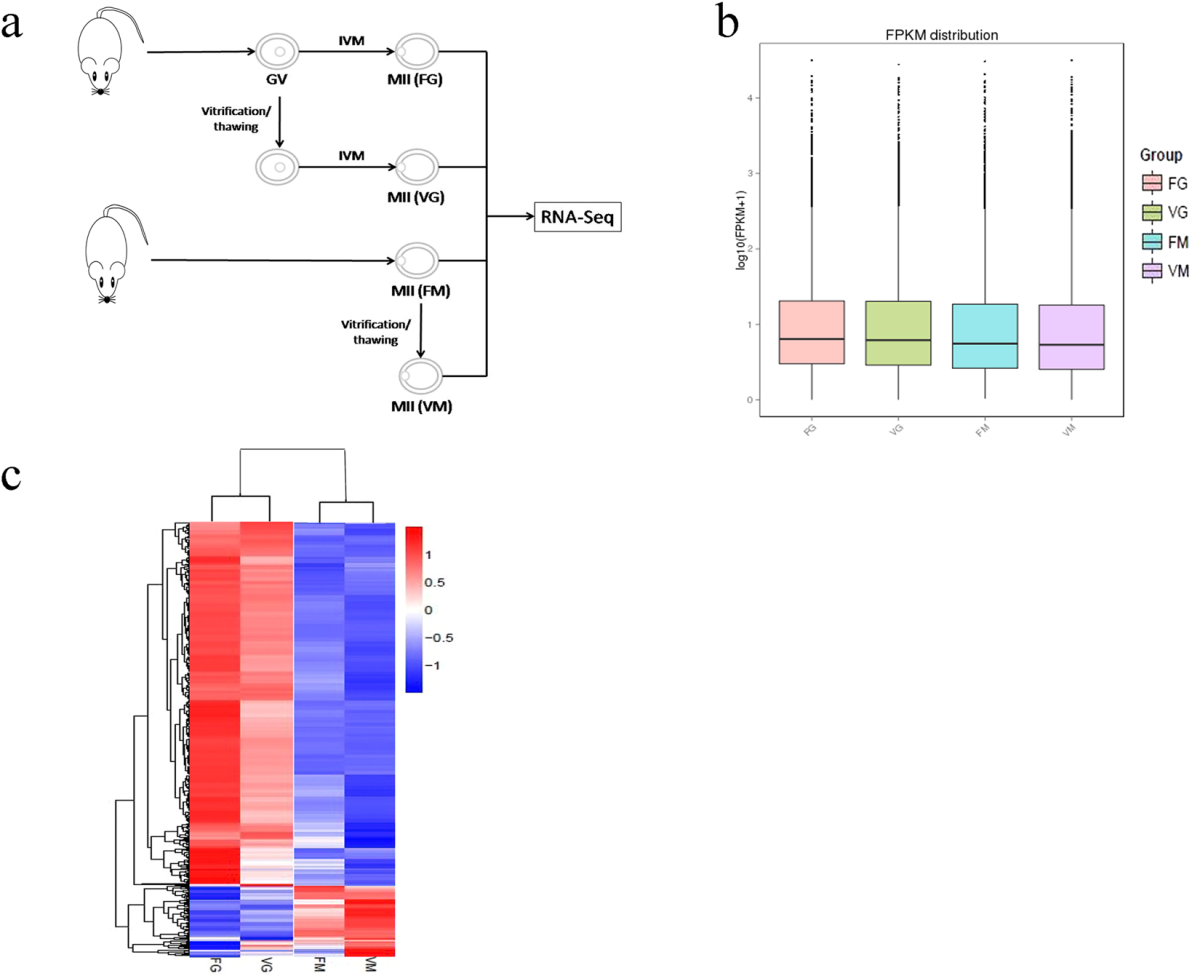
Experimental design and gene expression levels in the four groups.The figure (**a**) shows the experimental design. The boxplot (**b**) shows the gene expression level (log10 FPKM) in each sample. (**c**) shows the results of a clustering analysis of gene expression levels (FPKM) in each sample.

In this study, RNA-seq was used to analyse the transcriptomes of differently treated mouse MII stage oocytes to gain a better understanding of the transcriptome events in the oocytes after *in vitro* maturation and/or vitrification. The following four groups of mouse MII stage oocytes were used: 1. fresh GV stage oocytes matured *in vitro* (FG); 2. vitrified GV stage oocytes matured *in vitro* (VG); 3. fresh MII stage oocytes matured *in vivo* (FM); 4. vitrified MII stage oocytes matured *in vivo* (VM) (Fig. 1a). We also performed a Gene Ontology (GO) analysis of the differentially expressed genes. Our results contribute to the understanding of the transcriptional regulatory mechanisms of oocytes undergoing *in vitro* maturation and/or vitrification and offer a theoretical basis to further improve the efficiency of the *in vitro* maturation and/or vitrification of oocytes. This study also provided a transcriptional basis for using oocyte vitrification to preserve female fertility for those who may lose their ovarian function because of surgery, cancer treatment and premature menopause or who decide to postpone having children.

## Results

### Summary of RNA-Seq data quality

We performed RNA-seq to analyse the transcriptomes of MII stage mouse oocytes from different groups, including FG, VG, FM and VM (Fig. 1a). Approximately 532 million reads (82.99% of clean reads) mapped to the reference genome and 79.83% were uniquely mapped (Table S1). The expression of each transcript in each sample was measured as the expected number of fragments per kilobase of transcript sequence per millions base pairs sequenced (FPKM). Genes with values of FPKM >1 were considered to be genes expressed in this study. Boxplots of log10-transformed FPKM values for each replicate showed that the overall range and distribution of the FPKM values were consistent among the samples (Fig. 1b). R^2 values were between 0.889 and 0.978 for all three replicates within the four groups, thus indicating that there were no significant differences in gene expression among the biological replicates (Figure S1). These results indicated that our RNA-seq data was reliable, reproducible and of high quality. Cluster analysis also showed that differences occurred among the four samples, thus confirming that our RNA-seq data met the conditions for differential expression analysis (Fig. 1c).

### *In vitro* maturation influence the genes expression of mitochondrial membrane protein

**Figure 2:**
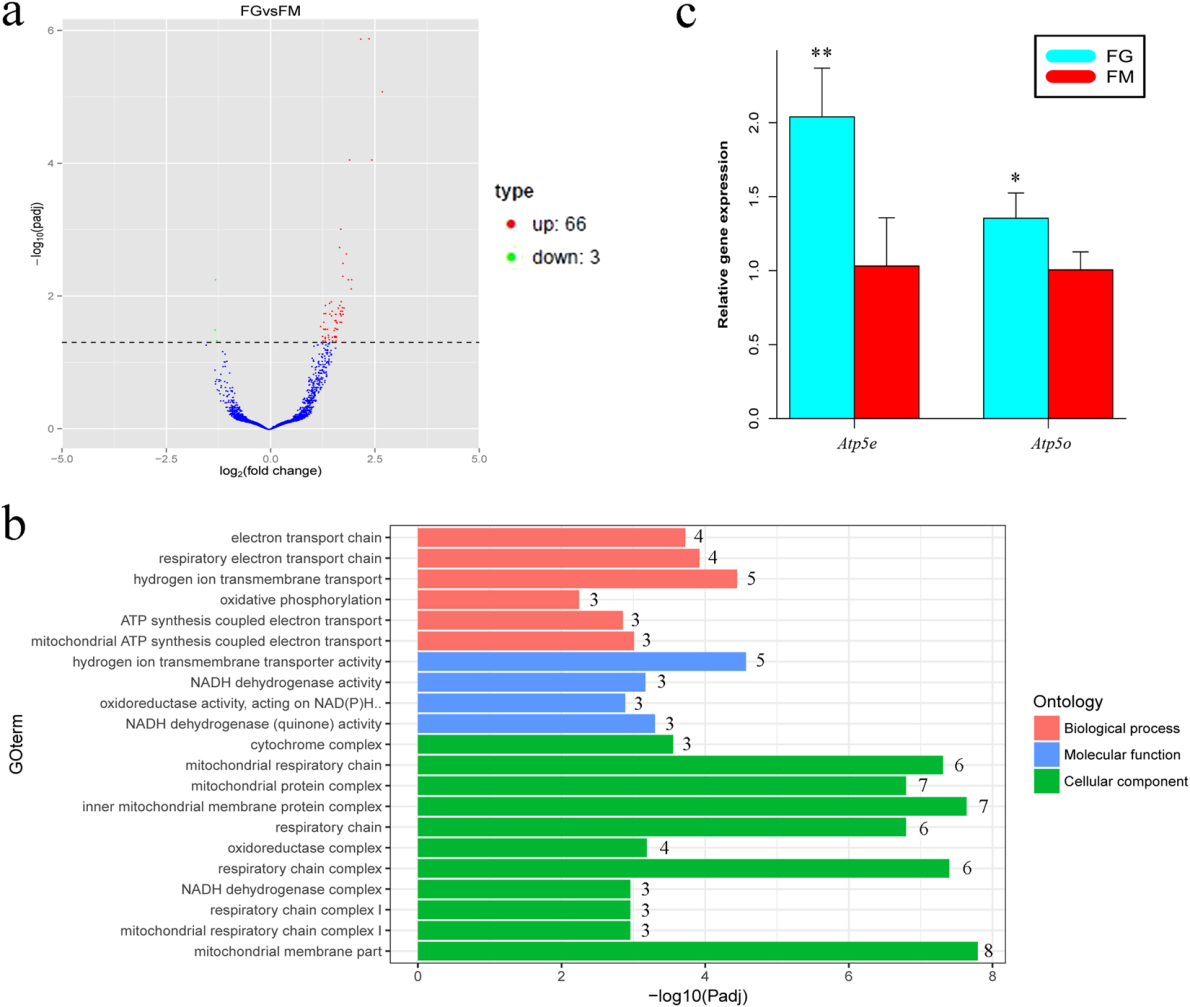
Oocytes matured *in vitro* influence the genes expression of mitochondrial membrane protein The Volcano plot (**a**) shows differentially expressed genes meeting the conditions of adjusted P-value < 0.05. Red dots represent the 66 genes up-regulated in FG vs. FM, green dots represent 3 genes down-regulated in FG vs. FM, and blue dots represent the genes that were not differently expressed in FG vs. FM. (**b**) we included the most significant GO categories affected by *in vitro* maturation (FG vs. FM) based on adjusted *p*-values. Red represents terms relating to biological processes, green represents terms relating to cellular components and blue represents terms relating to molecular function. The number of genes in each term is shown to the right of the pillar. (**c**) *Atp5e* and *Atp5o* were randomly selected and examined by qRT-PCR. The expression of these genes was consistent with the differential expression patterns observed in the RNA-seq data. Cyan represents FG group, red represents FM group.

To analyse the effects of *in vitro* maturation on gene expression in MII stage oocytes, we compared the gene expression profiles of FG with FM. Genes with FPKM >1 and adjusted p-value < 0.05 were considered to represent significant DEGs (differentially expressed genes) and that was also the case for similarly analysed data throughout this study. In total, 69 DEGs were identified (Table S2): 66 up-regulated and 3 down-regulated (Fig. 2a). GO enrichment analysis was performed to investigate which biological functions were important after IVM on 48 annotated DEGs of 69 total DEGs. “Mitochondrial membrane part” was the significant enrichment term in cellular components category, which involved 8 DEGs, including *Atp5e*, *Atp5o*, *Cox4i1*, *Cox7b*, *Ndufa5*, *Ndufb9*, *Ndufs7* and *Uqcrq*; “hydrogen ion transmembrane transport and activity” was the most significant enrichment term in biological processes category (Fig. 2b), which included 5 genes: *Atp5e*, *Atp5o*, *Cox4i1*, *Cox7b* and *Uqcrq* (Table S3). Interestingly, *Atp5e* and *Atp5o* were involved in both significant terms and occurred in the top 10 significant DEGs (Table S2). *Atp5e* and *Atp5o* were randomly selected and examined using qRT-PCR. The expression of these genes was consistent with the differential expression patterns observed in the RNA-seq data (Fig. 2c).

### Vitrification has negligible effects on transcriptome of oocytes

**Figure 3:**
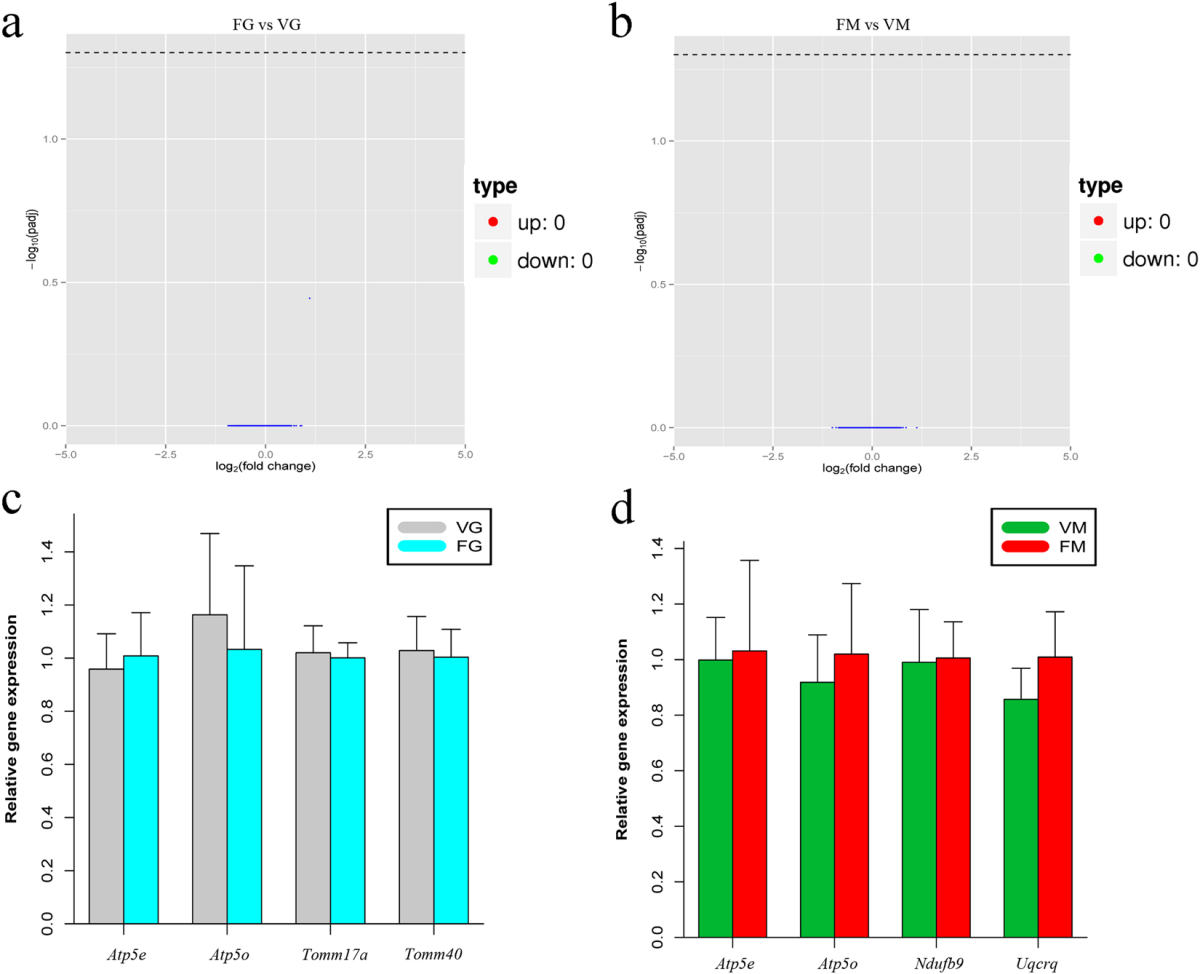
Vitrification has negligible Effects on transcriptome of oocytes. The Volcano plot (**a**) shows no DEGs identified in FG vs. VG, indicating that *in vitro* maturation of oocytes vitrified in the GV stage did not cause significant changes in transcriptome profiles when compared with those of fresh oocytes matured *in vitro*. The Volcano plot (**b**) shows that no DEGs were identified in FM vs. VM, indicating that vitrification did not change the transcriptome profile of MII stage oocytes matured *in vivo*. Atp5e, Atp5o, Timm17a and Tomm40 in FG vs. VG (**c**) and *Atp5e*, *Atp5o*, *Ndufb9* and *Uqcrq* in FM vs. VM (**d**) were randomly selected and examined with qRT-PCR. The expression of these genes was consistent with the differential expression patterns observed in the RNA-seq data. Cyan represents FG group, gray represents VG group, red represents FM group, green represents VM group.

This experiment was carried out to compare the gene expression profiles of FG vs. VG and FM vs. VM (Fig. 1a). No DEGs were identified between FG and VG, indicating that *in vitro* maturation of oocytes vitrified in the GV stage did not cause significant changes in the transcriptome profile compared with fresh oocytes matured *in vitro* (Fig. 3a). Additionally, there were no DEGs between FM and VM, indicating that vitrification did not change the transcriptome profile of MII stage oocytes matured *in vivo* (Fig. 3b). When some genes were randomly selected in DEGs of FG vs. VG and FM vs. VM and were examined with qRT-PCR, the results showed that the expression of these genes was consistent with the differential expression patterns observed in the RNA-seq data (Fig. 3c and d).

### The MII stage is more suitable for oocyte vitrification from the view of transcriptome

**Figure 4:**
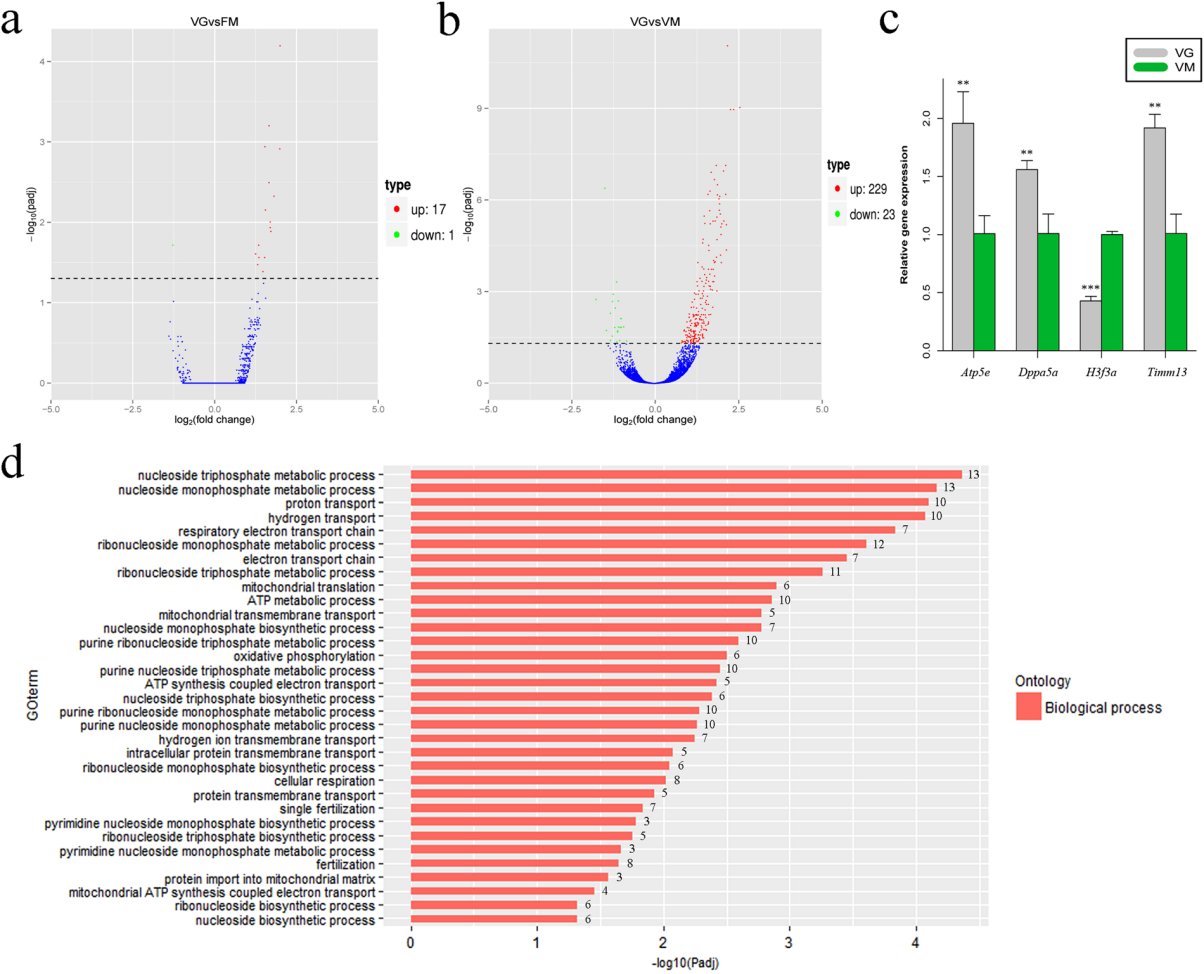
The transcriptome profiles have a significant difference in MII oocytes obtained from GV or MII vitrification. The Volcano plot (**a**) shows 18 DRGs were identified in VG vs. FM. Red dots represent 17 up-regulated genes, green dots represent 1 down-regulated gene, and blue dots represent the genes which were not differentially expressed in VG vs. FM. The Volcano plot (**b**) shows a total of 252 genes that were differentially expressed in VG vs. VM. Red dots represent 229 up-regulated genes, green dots represent 23 down-regulated genes, and blue dots represent the genes that were not differentially expressed in VG vs. VM. (**c**) *Atp5e*, *Dppa5a*, *H3f3a* and *Timm13* were randomly selected and examined by qRT-PCR. The expression of these genes was consistent with the differential expression patterns observed in the RNA-seq data. Gray represents VG group, green represents VM group. (**d**) The significant GO categories were related to biological process. The number of genes in each term is shown to the right of the corresponding bar.

Our results showed that no DEGs were found in FM vs. VM; however, 18 DEGs were identified in VG vs. FM, indicating that the transcriptome profile of oocytes vitrified in the MII stage was more similar to those of oocytes matured *in vivo* (Fig. 4a, Table S4). However, 252 DEGs were identified between VG and VM (Table S5), of which 229 were up-regulated and 23 were down-regulated (Fig. 4b). GO analysis of 212 annotated genes of the 252 total DEGs found that the most significant enrichment term is “inner mitochondrial membrane protein complex”, which has 22 genes, including Atp5o, Tomm40 and Timm13 (Figure S2a). In molecular function category, most terms correlated with NADH activity (Figure S2b). The term “mitochondrial transmembrane transport” was significantly enriched in biological processes category, which included 5 genes: *Tomm40*, *Timm13*, *Timm17a*, *Atp5o* and *Dnlz* (Fig. 4d, Table S6). The differentially expressed genes *Atp5e*, *Dppa5a*, *Haf3a*, *Timm13* in VG vs. VM and *Dppa5a* in VG vs. FM were randomly selected and examined by qRT-PCR; the expression of these genes was consistent with the differential expression patterns observed in the RNA-seq data (Fig. 4c, Figure S3).

### Mitochondrial membrane protein gene expression differs between *in vitro* maturation of fresh and vitrified GV stage oocytes

**Figure 5:**
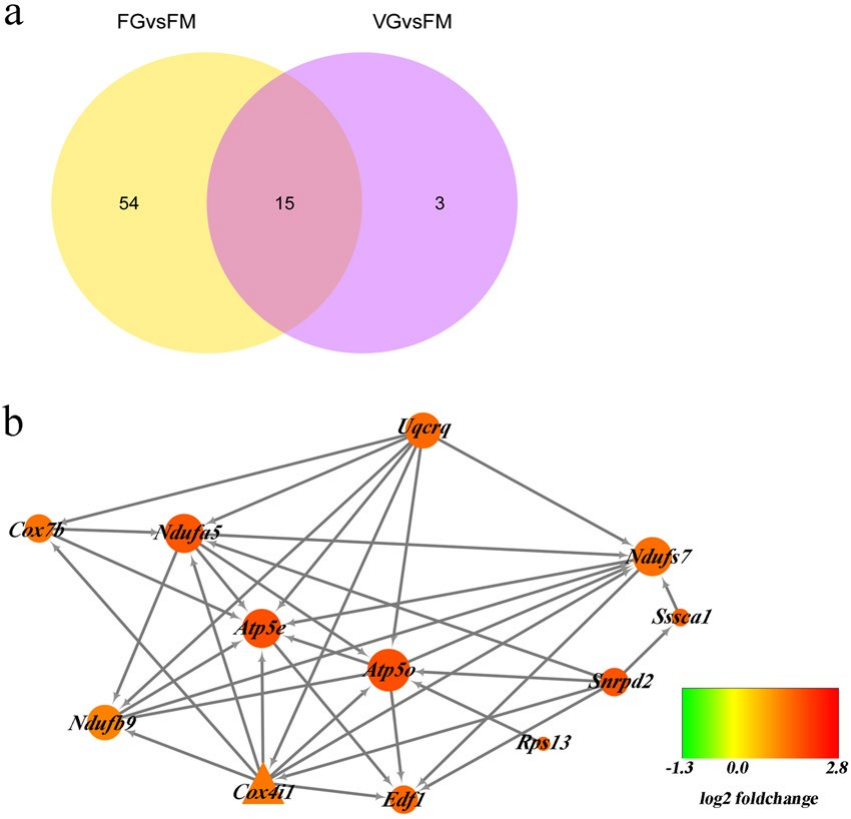
Mitochondrial membrane protein gene expression is different between in vitro maturation of fresh and vitrified GV oocytes. Venn diagram (**a**) shows 15 overlapping DEGs in FG vs. FM and VG vs. FM; 54 specific DEGs only occur in FG vs. FM, and 3 DEGs only occur in VG vs. FM. (**b**) Protein-protein interaction networks of the DEGs in FG vs. FM and VG vs. FM. **N**o interaction were identified between DEGs in VG vs. FM, however, interactions were identified between 12 DEGs in FG vs. FM. All of the 12 DEGs were up-regulated and were mitochondrial membrane protein genes. Among them, *Cox4i1* was the only DEGs that belong to the 15 overlapped DEGs. Circles represent specific genes in FG vs. FM, The triangle represents the only interacted DEGs (*Cox4i1*) which belong to the 15 overlapped DEGs (Fig. 5a). Size of the circle is proportional to the number of DEGs interacted with it, the color represents Log2FoldChange in expression levels of differentially expressed genes between FG and FM. The network indicates that *Atp5e* and *Atp5o* play key roles in the PPI network.

Only 18 DEGs were identified in VG vs. FM, which is lower than the number of DEGs in FG vs. FM, which have 69 DEGs. There were 15 overlapped DEGs between FG vs. FM and VG vs. FM, 54 specific DEGs in FG vs. FM and 3 specific DEGs in VG vs. FM. It seems that VG did behave more transcriptomically like the oocytes matured *in vivo* (Fig. 5a). Protein-protein interaction (PPI) analysis was performed to identify the interactions between DEGs. No interaction were identified between DEGs in VG vs. FM, however, interactions were identified between 12 DEGs in FG vs. FM. All of the 12 DEGs were up-regulated and were mitochondrial membrane protein genes. Among them, *Cox4i1* was the only DEGs that belong to the 15 overlapped DEGs. Additionally, the ATP synthase genes *Atp5e* and *Atp5o* were in the key position of the PPI network (Fig. 5b). These results indicated that the mitochondrial membrane protein gene expression was different between *in vitro* maturation of fresh and vitrified GV stage oocytes when compared to FM.

## Discussion

RNA-seq does not require pre-designed probes and can detect and quantify entire transcripts, including unknown transcripts^19^; it therefore greatly facilitates the study of the influence of *in vitro* maturation and/or vitrification on mouse oocyte gene expression profiles.

In this study, most of the DEGs were up-regulated after IVM, which is in accordance with previous studies showing that most DEGs were up-regulated in human oocytes matured *in vitro* compared with those matured *in vivo* ^4,20^. However, the suboptimal quality of the material (oocytes failed to fertilize in most cases) and uncontrolled variables in those studies make it difficult to interpret the data. To avoid these limitations, we used mouse oocytes to analyse the influence of IVM on oocytes. In our results, *Atp5e* and *Atp5o* were in the top 10 significant DEGs in FG vs. FM and were also in the most significantly enriched GO term of “mitochondrial membrane part” and “hydrogen ion transmembrane transport” (Table S2, Fig. 2b), which indicated that *Atp5e* and *Atp5o* may play an important role in the *in vitro* maturation of oocytes. *Atp5e* and *Atp5o* were correlated with ATP synthase. *Atp5e* encodes the ε subunit, which is an essential subunit in the biosynthesis and assembly of the F1 portion of ATP synthase^21^. *ATP5o*, located in the stalk of the ATP synthase complex, appears to connect the catalytic core (F1 subunit) and the membrane proton channel (F0 subunit)^22^. This result may explain why the ATP content was significantly increased during the *in vitro* maturation of mouse oocytes^23^. It has been reported that oocytes with higher concentration of ATP have significantly higher fertilization and blastocyst rates^24^. Thus, *Atp5e* and *Atp5o* may play an important role in increasing the ATP level during the *in vitro* maturation of oocytes and may be important biomarkers for oocytes matured *in vitro*.

It seems the influence of vitrification on the transcriptome is negligible, as no DEGs were found between the FG and VG (Fig. 3a) or FM and VM groups (Fig. 3b). These findings differ from previous studies that reported decreased mRNA expression in vitrified human MII stage oocytes as assessed using traditional reverse transcription-PCR^25^ and DNA microarray^26^. However, many of the oocytes they used were unfertilized MII stage oocytes after ICSI, and the time of oocyte collection varied. Recently, a study using RNA-seq showed that vitrification of bovine oocytes matured *in vitro* exhibited changes in the expression of some genes; however, the oocytes they used came from bovine ovaries obtained from a local abattoir^27^, and the different genetic background among these oocytes should not be ignored. In this study, oocytes obtained from CD-1 mice (all of the same genetic background), could better explore the influence of vitrification, and no significant difference in the transcription of oocytes after vitrification was found. To some extent, these results are consistent with a previous report that vitrified oocytes from the same cohort of donor oocytes (oocytes from same donor(s)) and showed no statistically significant differences in fertilization, embryo quality or clinical results when compared with fresh oocytes^28–30^.

However, it seems that oocytes vitrified at the GV or MII stage have different transcriptome profiles. In this study, we found that the largest transcriptional difference between VG and VM was mitochondrial function, especially NADH activity (Figure S2b). We found that *Ndufa2*, *Ndufa5*, *Ndufa7*, *Ndufb7*, *Ndufb9* and *Ndufs7* were enriched in all of the terms related to NADH activities. All of these genes were up-regulated and related to NADH dehydrogenase activity, and are in accordance with previous results showing that the ratio of FAD/NAD(P)H is significantly higher in vitrified human oocytes^10^. An increased ratio of FAD/NAD(P)H suggests a decreased ATP level in vitrified human oocytes^9,11^, which can significantly affect spindle integrity and chromosome alignment^10,12^. *In vitro* matured porcine oocytes obtained from the GV vitrification group exhibited higher abnormal configurations of spindles and chromosomes than oocytes from the MII vitrification group^31^.

In addition, most of the DEGs identified in VG vs. VM were related with mitochondria and their products must undergo transmembrane transport to the mitochondria. *Tomm40*, an important gene for a channel-forming protein involved in the biological process of “mitochondrial transmembrane transport” (Fig. 4d), is essential for the import of protein precursors into the mitochondria. The deletion of *Tomm40* results in the cytosolic accumulation of unprocessed mitochondrial precursors and subsequent lethality^32^. Thus, *Tomm40* may be the most promising candidate gene for oocyte viability at different vitrification stages. There have been many reports regarding the various viabilities of oocytes when vitrified in different stages^33,34^; however, the molecular mechanism of such variability is still unclear. Our results showed no DEGs in FM vs. VM and 18 DEGs in VG vs. FM (Fig. 3b and Fig. 4a), indicating the transcriptome profile of oocytes vitrified in the MII stage was closer to oocytes matured *in vivo*.

Mitochondrial membrane protein gene expression was different between fresh and vitrified GV stage oocytes following *in vitro* maturation. We noted that the number of DEGs in VG vs. FM was lower compared with DEGs in FG vs. FM (Fig. 5a). It appears that *in vitro* maturation allows vitrified GV stage oocytes to recover and behave more transcriptomically like the oocytes matured *in vivo*. The developmental competence of the vitrified-thawed GV stage oocytes (VG), however, was lower than in the *in vitro* matured oocytes (FG)^34^. Additionally, the abnormal configuration of spindles and chromosomes in mouse cryo-MII group (vitrified at the GV stage, warmed and matured *in vitro*, VG) oocytes (78.9 and 84.2%) was significantly higher than *in vitro*-MII (oocytes matured *in vitro*, FG) oocytes (45.0 and 50.0%, *P* < 0.05)^33^. *Atp5e* and *Atp5o*, which both occupy key positions in the PPI network, were up-regulated in FG vs. FM; however, *Atp5e* and *Atp5o* were not up-regulated in VG vs. FM. This finding provides a new explanation for previous results in which the ATP content was significantly lower during *in vitro* maturation of vitrified GV stage oocytes compared with fresh GV stage oocytes^35^. Low ATP content may result in decreased rates of normal spindle formation^12^ which is also correlated with a high aneuploidy rate. Aneuploidy rates in oocytes matured from vitrified-thawed GV stage oocytes (VG) were significantly higher than from *in vitro* matured oocytes (FG) (22.9% vs. 5.8%, respectively; *P* < 0.05)^34^.

## Conclusions

We used RNA-seq and *in silico* pathway analysis to identify DEGs that may be involved in oocyte viability after *in vitro* maturation and/or vitrification. Our results showed the following: 1) *in vitro* maturation influences the mitochondrial membrane protein gene expression, especially those associated with ATP synthase (*Atp5e* and *Atp5o*); 2) oocyte vitrification caused minor or no changes in MII stage oocyte transcriptome profiles; and 3) the MII stage is more suitable for oocyte vitrification from the viewpoint of the transcriptome. This study can contribute to the understanding of the transcriptional regulatory mechanisms, and offer a theoretical basis to further improve the efficiency, of oocytes undergoing *in vitro* maturation and/or vitrification.

## Materials and Methods

### Ethics statement

The use of the animals and the experimental procedures were approved by the Animal Care Committee at China Agricultural University. The experiment was conducted at China Agricultural University. All methods were carried out in accordance with the approved guidelines.

### Reagents

All chemicals and media were purchased from Sigma Chemical Co. (St. Louis, MO, USA), unless otherwise indicated.

### Oocyte collection

Oocytes were collected from 8- to 10-week-old CD-1 mice (Vital River Laboratory Animal Technology Co. Ltd. China) as described previously^34^. The GV oocytes were collected from 8- to 10-week-old mice by ovarian puncture, and then the oocytes were collected by mouth pipette. For *in vitro*-matured MII oocytes, GV oocytes were cultured in M16 medium (foetal bovine serum 50 µL/mL, follicle stimulating hormone 1 mg/mL, luteotropic hormone 500 µg/mL, epidermal growth factor 5 µg/mL and Sodium pyruvate 23 mmol/L) for 18 h, and only oocytes with polar body extrusion were collected. For *in vivo*-matured MII oocyte retrieval, mice were superovulated with 10 IU (intraperitoneal) equine chorionic gonadotropin (eCG; Ningbo Hormone Products Co, China), which was followed 48 hours later by an injection of 10 IU of human chorionic gonadotropin (hCG; Ningbo Hormone Products Co. China). Cumulus-oocyte complexes (COCs) were collected from oviducts at 13 hours after hCG treatment and recovered in M2 medium. Cumulus cells were dispersed by hyaluronidase (300 IU/mL) for 3–5 minutes in M2 medium. Ten mice were used in each case.

### Oocyte cryopreservation

#### Vitrification solutions

Pretreatment solution contained 10% dimethylsulfoxide (DMSO) and 10% ethylene glycol (EG) in PBS medium. Vitrification solution (EDFS30) contained 15% DMSO (v:v), 15% EG(v:v), 30% Ficoll (w:v) and 0.5 M sucrose in PBS medium.

#### Vitrification and warming

Oocytes were vitrified in EDFS30 by the open-pulled straws (OPS) method^36^. First, oocytes were pretreated in pretreatment solution for 30 seconds, transferred to vitrification solution in the narrow end of the OPS, and held for 25 seconds. Then, the straws were immediately plunged into liquid nitrogen (LN2). For thawing, the oocytes were rinsed in 0.5 M sucrose for 5 minutes, then rinsed three times in M2 medium. Over 100 GV oocytes and 100 MII oocytes were collected respectively after thawing.

#### RNA quantification and qualification

Oocytes were incubated in M2 containing 0.5% pronase for 2–3 min to remove their ZP (zona pellucida). ZP-free oocytes were carefully washed several times with M2. The 100 ZP-free oocytes per group were collected via mouth-pipette and stored in RNA lysis buffer from a SMART-Seq™ v4 Ultra™ Low Input RNA Kit (Clontech laboratories, Inc., CA, USA). The purified total RNA was stored in nuclease-free water, then used for first-strand synthesis. RNA concentration was measured using a Qubit® RNA Assay Kit in a Qubit® 2.0 Fluorimeter (Life Technologies, CA, USA). The results of RNA concentration were shown in Table S1.

#### cDNA amplification and library preparation for transcriptome sequencing

First-strand cDNA (from total RNA) was synthesized according to the SMART-Seq™ v4 Ultra™ Low Input RNA Kit protocol. The PCR-amplified cDNA was purified using AMPure XP beads, then 1 μl cDNA was validated using an Agilent 2100 Bioanalyzer.

cDNA samples were sheared with a Covaris system before library preparation. Sequencing libraries were generated using a NEBNext® Ultra™ DNA Library Prep Kit for Illumina® (NEB, USA) according to the manufacturer’s recommendations. In short, the workflow included conversion of sheared DNA into blunt ends, adenylation of the DNA fragments 3′ ends, ligation of index-coded adapters (The Index Primers were shown in Table S7) and PCR amplification. Finally, PCR products were purified (AMPure XP system) and library quality was assessed on an Agilent Bioanalyzer 2100 system (effective concentration of the cDNA libraries >2 nM). These libraries were sequenced using an Illumina Hiseq platform with 150 bp paired-end sequencing at the Novogene Bioinformatics Institute (Beijing, China).

### Quality control

Raw data (raw reads) in fastq format were first processed with in-house Perl scripts. In this step, clean data (clean reads) were obtained by removal of reads containing adapter sequences, reads containing ploy-N and low quality reads from the raw data. All the downstream analyses were based on clean, high-quality data.

### Reads mapping to the reference genome

The reference genome and gene model annotation files were downloaded directly from the genome website (ftp: //ftp.ensembl.org/pub/release-79/fasta/mus_musculus/dna/ and ftp: //ftp.ensembl.org/pub/release-79/gtf/mus_musculus/). The reference genome index was built using Bowtie v2.2.3, and clean paired-end reads were aligned to the reference genome with TopHat v2.0.12.

### Quantification of gene expression levels

HTSeq v0.6.1 was used to count the read numbers mapped to each gene. Then, the FPKM of each gene was calculated on the basis of the length of the gene and read counts mapped to this gene^37^.

### Differential expression analysis

Differential expression analysis was performed using the DESeq R package (1.18.0). DESeq provides statistical routines for determining differential expression in digital gene expression data by using a model based on a negative binomial distribution. The resulting P-values were adjusted using Benjamini and Hochberg’s approach for controlling the false discovery rate. Genes with an adjusted P-value < 0.05 as calculated by DESeq were considered differentially expressed.

### GO analysis of differentially expressed genes

Differentially expressed genes were called if they satisfied the conditions adjusted P-value < 0.05. Ensemble gene IDs were translated into official gene symbol IDs with BioMart. An analysis of the enrichment of differentially expressed genes was conducted using ClueGO of the Cytoscape software, and P-Values were corrected by the method of Benjamini-Hochberg. A corrected P-value ≤ 0.05 was considered to indicate significant gene enrichment.

### PPI analysis of differentially expressed genes

PPI analysis of differentially expressed genes was performed on the basis of the STRING protein interaction database (http://string-db.org/). Visual analysis of PPI network data files of differentially expressed genes were created with the Cytoscape software.

### qRT-PCR verification

RNA was extracted from 60 oocytes per group utilizing TRIzol reagent (Invitrogen, USA) according to the manufacturer’s instructions. Approximately 0.05 μg RNA was used to synthesize cDNA with an Ambion Cells-to-Complementary DNA II kit (Life Technologies, Inc., Grand Island, NY, USA). qRT-PCR was performed as described previously^38^ to quantify the mRNA levels of *Atp5e*, *Atp5o*, *Ndufb9*, *Uqcrq*, *Timm17a*, *Dppa5a*, *H3f3a*, *Timm13* and *Tomm40*. *Gapdh* was used as a reference gene. Quantitative realtime PCR was performed using the CFX96TM Real-Time PCR Detection System (Bio-Rad) under standard conditions. The expression levels were calculated using the 2^−DDCt^ method described previously^39^. The primers are shown in Table S8. Each experiment was repeated at least 3 times. All of the data are presented as the means ± SD. The data were analysed by one-way ANOVA using SPSS 20.0 software and p < 0.05 was considered significant.

## Electronic supplementary material

Supplementary Information

Supplementary Table 1

Supplementary Table 2

Supplementary Table 3

Supplementary Table 4

Supplementary Table 5

Supplementary Table 6

Supplementary Table 7

Supplementary Table 8
