# Supplementary material for: RNA-Seq transcriptome profiling of mouse oocytes after in vitro maturation and/or vitrification
Source: Sci Rep. 2017 Oct 16;7:13245. doi: 10.1038/s41598-017-13381-5 (PMC5643491; doi:10.1038/s41598-017-13381-5)
Supplement: Supplementary file 1 — Supplementary Information [file 41598_2017_13381_MOESM1_ESM.doc]

**RNA-Seq transcriptome profiling of mouse oocytes after *in vitro* maturation and/or vitrification**

Lei Gao^1^, Gongxue Jia^2^, Ai Li^3^, Haojia Ma^1^, Zhengyuan Huang^1^, Shien Zhu^1^, Yunpeng Hou ^4^ and Xiangwei Fu ^1^

1. National Engineering Laboratory for Animal Breeding and Key Laboratory of Animal Genetics, Breeding and Reproduction, Ministry of Agriculture, College of Animal Science and Technology, China Agricultural University, Beijing 100193,P.R China;

2. Key Laboratory of Adaptation and Evolution of Plateau Biota, Northwest Institute of Plateau Biology, Chinese Academy of Sciences, Xining 810001, P.R China;

3. Institute of Animal Sciences, Chinese Academy of Agricultural Sciences, Bejing 100193, P.R China

4. State Key Laboratory for Agrobiotechnology, College of Biological Sciences, China Agricultural University, Beijing 100193, P.R China

Lei Gao and Gongxue Jia contributed equally to this work.

Corresponding author: Tel. /fax: +86 10 62731767. E-mail addresses: xiangweifu@126.com (X. Fu)


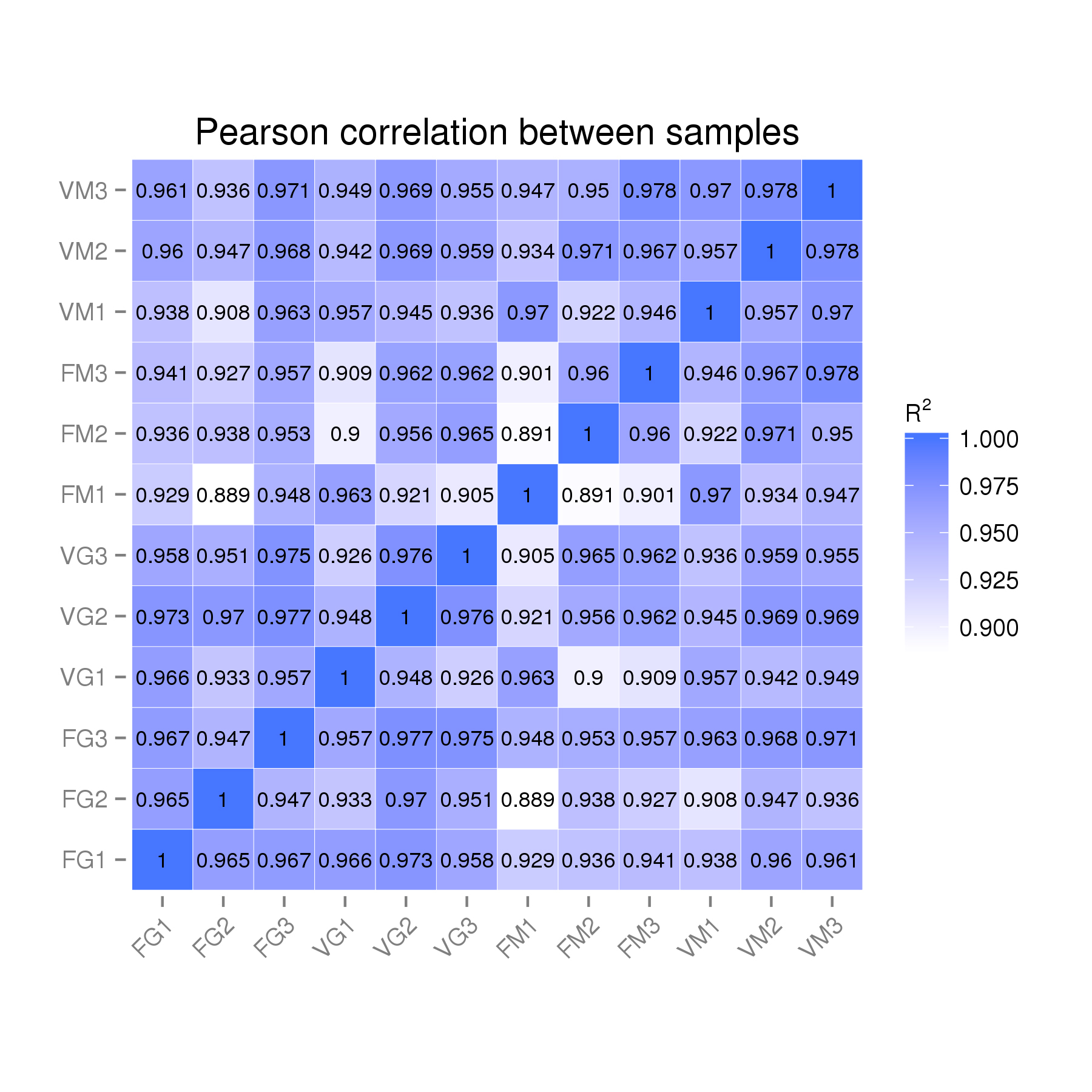


**Supplementary Figure 1 Correlation plot for gene expression of different sample**

The R^2^ values were between 0.889 and 0.978 for all the three replicates within four groups, indicating that there are no significant differences in gene expression among the biological replicates and the technical variation is reasonably low.

**
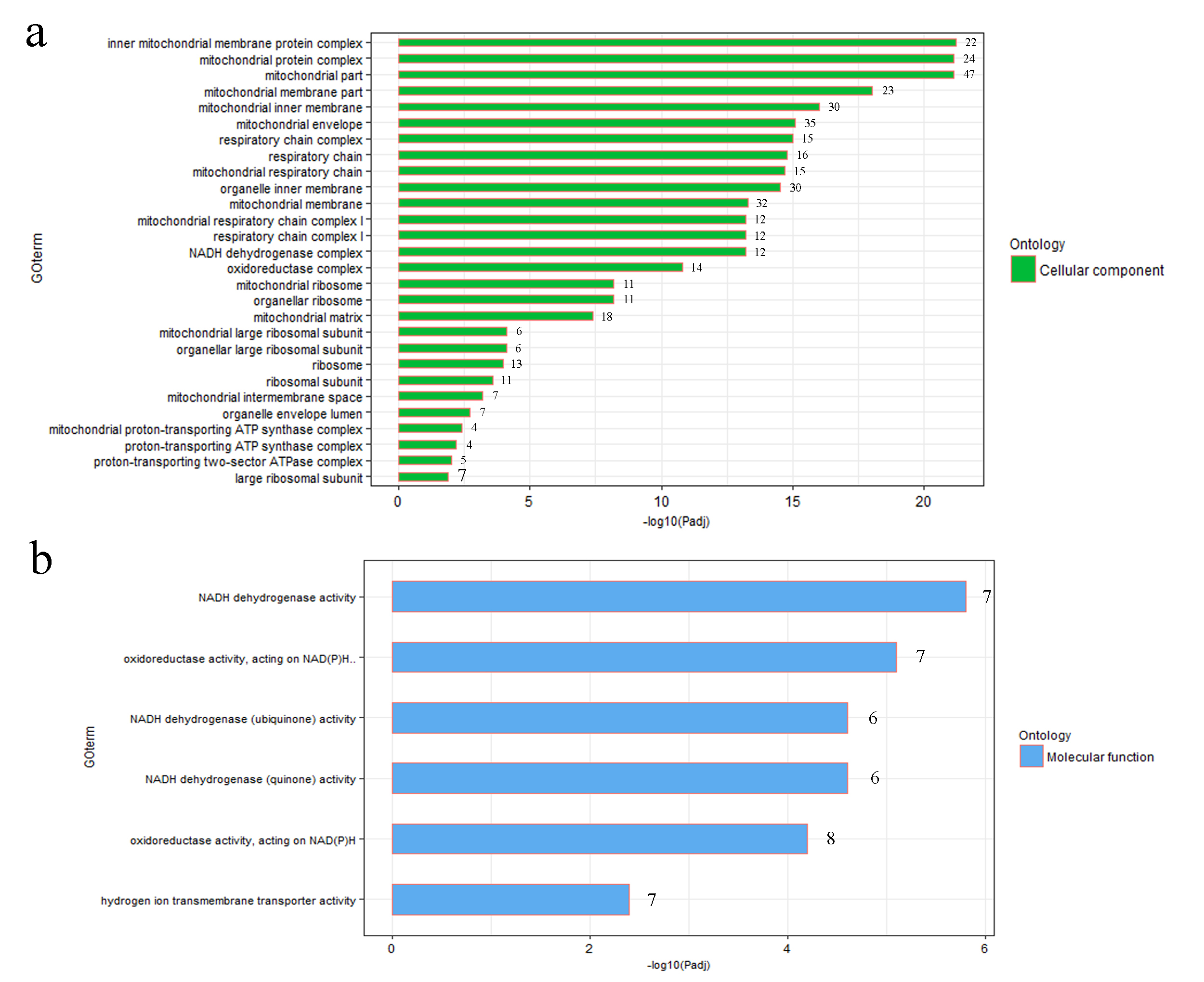
**

**Supplementary Figure 2 GO analysis of the DEGs in VG vs. VM.**

(a) The most significant GO categories in cellular component. (b) The most significant GO categories in molecular function. The number of genes in each term is shown in the right of the corresponding bar.


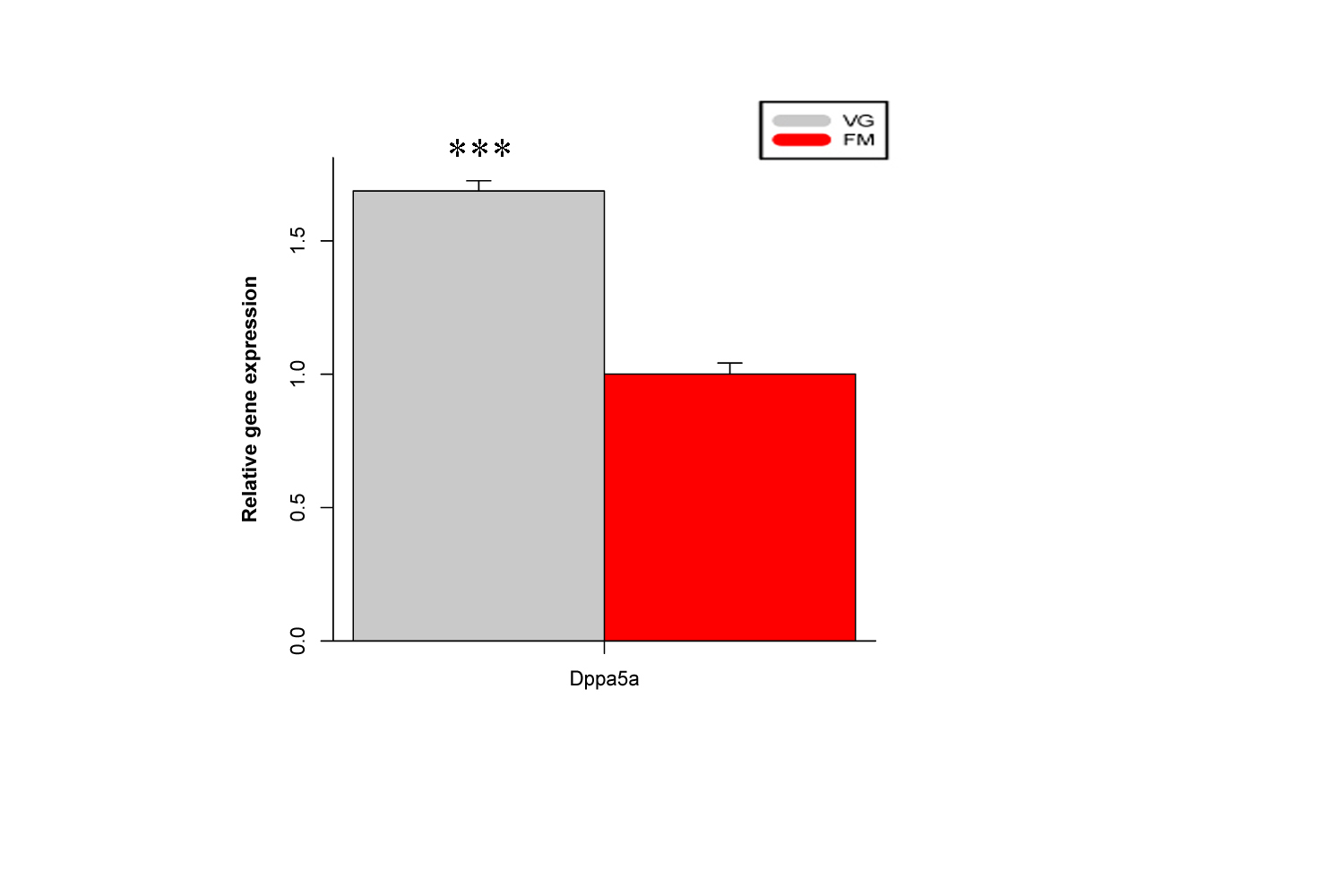


**Supplementary Figure 3**

Dppa5a was randomly selected in VG vs. FM and examined with qRT-PCR.The expression of these genes was consistent with the differential expression patterns observed in the RNA-seq data.

**Supplementary Table 1 Reads mapping summary**

**Supplementary Table 2 differently expressed genes between FG and FM**

**Supplementary Table 3 Gene Ontology analysis of differently expressed genes between FG and FM**

**Supplementary Table 4 differently expressed genes between VG and FM**

**Supplementary Table 5 differently expressed genes between VG and VM**

**Supplementary Table 6 Gene Ontology analysis of differently expressed genes between VG and VM**

**Supplementary Table 7 Index primers**

**Supplementary Table 8 Primer sequences**
